# Hepatotoxicity induced by horse ATG and reversed by rabbit ATG: a case report

**DOI:** 10.1186/1752-1947-1-35

**Published:** 2007-06-28

**Authors:** Khalid A Al-Anazi, Mahmoud D Aljurf, Fahad Z Al-Sharif, Hamad M Al-Omar, Ahmed Alami, Fayyaz Farooq

**Affiliations:** 1 Section of Adult Hematology and Hematopoietic Stem Cell Transplant, King Faisal Cancer Centre, King Faisal Specialist Hospital and Research Centre, P.O. Box: 3354, Riyadh 11211, Saudi Arabia; 2 Department of Pharmacy Services, King Faisal Specialist Hospital and Research Centre, P.O. Box: 3354, Riyadh 11211, Saudi Arabia

## Abstract

**Background:**

The use of antilymphocyte agents has improved patient and graft survival in hematopoietic stem cell and solid organ transplantation but has been associated with the development of short-term toxicities as well as long-term complications.

**Case presentation:**

We report a young female with Fanconi anemia who received antithymocyte globulin as part of the conditioning regimen prior to her planned allogeneic hematopoietic stem cell transplant at King Faisal Specialist Hospital and Research Centre in Riyadh. She developed sudden and severe hepatotoxicity after receiving the first dose of horse antithymocyte globulin, manifested by marked elevation of serum transaminases and mild elevation of serum bilirubin level. Immediately after withdrawal of the offending agent and shifting to the rabbit form of antithymocyte globulin, the gross liver dysfunction started to subside and the hepatic profile results returned to the pre-transplant levels few weeks later. The patient had her allogeneic hematopoietic stem cell transplant as planned without any further hepatic complications. After having a successful allograft, she was discharged from the stem cell transplant unit. During her follow up at the outpatient clinic, the patient remained very well and no major complication was encountered.

**Conclusion:**

Hepatotoxicity related to the utilization of antithymocyte globulin varies considerably in severity and may be transient or long standing. There may be individual or population based susceptibilities to the development of side effects and these adverse reactions may also vary with the choice of the agent used. Encountering adverse effects with one type of antithymocyte agents should not discourage clinicians from shifting to another type in situations where continuation of the drug is vital.

## Background

Antithymocyte globulin (ATG) has been used effectively and safely in the treatment of aplastic anemia, in the conditioning regimens of hematopoietic stem cell transplant (HSCT), in the treatment of acute graft versus host disease (GVHD) and in the prevention and treatment of acute rejection in solid organ transplantation [1-5].

Rabbit ATG is a purified polyclonal immunoglobulin G (IgG) prepared from the plasma of healthy rabbits hyperimmunized with a human T cell line [4,6]. The high specific activity and antibody content imply the need for lower doses with reduced side effects in comparison to the other antilymphocyte agents [4]. Like all other immunosuppressive agents, ATG has several adverse effects that include variable degrees of hepatotoxicity [2-4].

Recent advances in immunosuppressive therapy have resulted in significantly improved patient and graft survival after solid organ transplantation [7]. However, the increased utilization of immunosuppressive agents has brought special attention to specific toxicities associated with the use of these drugs [7]. As new agents are developed with narrow therapeutic windows, it will be essential to identify specific drug toxicities and to develop preventive and management therapeutic strategies [7].

## Case presentation

A 23 years old Saudi female, with no previous medical illnesses, was diagnosed to have Fanconi anemia (FA) at ARAMCO hospital in Dhahran in May 2002. Since then she became blood transfusion dependent, requiring 2 units of packed red blood cells every 6 to 8 weeks. However, the patient never received androgen therapy. After finding a healthy and an HLA identical sibling donor, the patient was transferred to King Faisal Specialist Hospital and Research Centre (KFSH&RC) in Riyadh for allogeneic HSCT. On 16/5/2005, she was admitted to the HSCT unit. She was totally asymptomatic and her physical examination revealed: pallor and pigmentation of the buccal mucosa but no jaundice, cyanosis, leg oedema or external lymphadenopathy. Her chest was clear, there were no murmurs or added heart sounds, no abdominal tenderness or palpable organomegaly and no neurological deficit. Blood counts were as follows: WBC: 1.83 × 10^9^/L, Hb: 83 g/L, PLT: 52 × 10^9^/L. Bone marrow examination showed a hypocellular marrow without any abnormal cell collection and a normal cytogenetic analysis. The renal and the coagulation profiles were within normal limits. The liver function tests were normal apart from a slight elevation of the serum level of alanine transaminase (ALT):78 U/L. The serum ferritin level was 850.8 μgram/litre [normal range: 13–150 μg/L]. Hepatitis serology and viral screens were negative. Ultrasound of the abdomen showed no focal liver abnormality. Chest radiograph, electrocardiogram and echocardiogram were all within normal limits.

**Figure 1:**
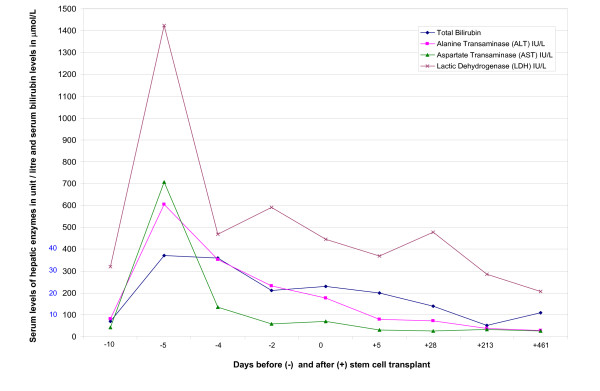
Shows the results of the liver function tests throughout the period of follow up. Days before (-) and after (+) stem cell transplant. Serum levels of hepatic enzymes in units/litre and serum bilirubin levels in μmol/litre.

On day-6 HSCT, the patient was commenced on a pediatric conditioning protocol composed of: horse ATG 40 mg/m^2 ^IV over 10 hours on days: -6, -4 and -2 HSCT and 20 mg/m^2 ^IV over 10 hours on days: +2, +4, +6, +8, +10 and +12 HSCT in addition to cyclophosphamide: 5 mg/Kg IV over 30 minutes on days: -5, -4, -3 and -2 HSCT. The patient was also given cyclosporin-A: 2.5 mg/kg IV twice daily as GVHD prophylaxis as well as infection prophylaxis in the form of acyclovir, trimethoprim-sulphamethoxazole and fluconazole. The patient received ATG premedications and she encountered no immediate reactions to the first dose of horse ATG. On day-5 HSCT, the patient was totally asymptomatic and her physical examination revealed no new abnormality but her liver function tests became severely disturbed: total bilirubin: 37 μmole/litre [normal range: 0–22 μmole/L], ALT: 604 unit/litre [normal range: 0–50 U/L], aspartate transaminase (AST): 708 U/L [normal range: 0–40 U/L] and lactic dehydrogenase (LDH): 1424 U/L [normal range: 70–250 U/L] (Figure 1). The horse ATG treatment was stopped, the planned dose of cyclophosphamide was held and fluconazole prophylaxis was discontinued temporarily to prevent further deterioration in the hepatic profiles. On day-4 HSCT, the patient remained clinicially stable and her elevated serum bilirubin and liver enzymes decreased by approximately 50%. The horse type of ATG was replaced by the rabbit form of ATG: 10 mg/m^2 ^IV over 10 hours on days: -4, -3 and -2 HSCT and 7 mg/m^2 ^IV over 10 hours on days: +2, +4, +6, +8, +10 and +12 HSCT. On day -3 HSCT, the patient remained clinically stable and her hepatic profiles improved further. Thereafter, the patient had progressive improvement in her liver function tests despite the continuation of rabbit ATG therapy (Figure 1). On day-1 HSCT, she received the dose of cyclophosphamide which was due 4 days earlier. One day later, the patient received her allograft without any complications and she was resumed on fluconazole prophylaxis. In the early post-transplant period, the patient developed grade II mucositis treated with PCA (patient controlled analgesia) tramadol and antiseptic mouth care in addition to aspiration pneumonia requiring artificial ventilation for 48 hours. No viral infections, acute GVHD or veno-occlusive disease of the liver were encountered during this hospitalization. The patient engrafted her leucocytes on day +25 and her platelets on day +16 HSCT. After the recovery of her blood counts and the discontinuation of the intravenous medications, the patient was discharged on day +28 HSCT on infection prophylaxis including trimethoprim-sulphamethoxazole and acyclovir till day +30 HSCT as well as cyclosporin-A 100 mg orally twice daily. Thereafter the patient had regular follow up at the HSCT outpatient clinic. Subsequently, she developed chronic GVHD of the liver which was treated with cyclosporin-A, prednisone and mycophenolate mofetil for a total duration of 8 months. The chimerism studies done on days +100 and +365 HSCT showed evidence of a successful engrafment as both the lymphoid and the myeloid cells were 100% donor type. The patient was last seen at the outpatient clinic on 01/10/2006. She was totally asymptomatic and her physical examination revealed no abnormality. Her blood indices were as follows: WBC: 10.6 × 10^9^/L, Hb: 122 g/L, PLT: 244 × 10^9^/L. Her renal and coagulation profiles were normal. The liver function tests were all within normal limits (Figure 1). The patient was given no new medication and she was given a new appointment for follow up.

## Discussion

Fanconi anemia is a genetic disorder associated with diverse congenital abnormalities, progressive bone marrow failure and an increased risk of leukemia and other cancers [8]. Allogeneic HSCT has been found to be an effective therapy for FA patients having a matched sibling donor [8-12]. Various pre-transplant conditioning protocols have been employed including the following in various combinations: low dose cyclophosphamide, ATG, radiotherapy (total body irradiation or limited field radiotherapy), fludarabine and busulphan [8-12]. Cyclosporin-A, methotrexate, daclizumab and even ATG have also been used in the GVHD prophylaxis [8-12]. Increased survival in FA patients undergoing allogeneic HSCT is associated with: younger age of the allograft recipients, having higher platelet counts in the pre-transplant period, the use of low dose cyclophosphamide and limited field radiotherapy in the pre-transplant conditioning protocols and the use of cyclosporin-A in the GVHD prophylaxis [8,9,11].

ATG is composed of immunoglobulin G (IgG) fraction of sera from rabbits or horses immunized with human thymocytes or T-cell lines [1]. ATG is a useful alternative to HSCT in selected patients with aplastic anemia [13]. It has the following advantages in comparison to HSCT: the costs are lower, the need for HLA-identical HSCT donors is abolished, the non-existence of GVHD and the possibility of treating patients more than 30 years old even those pre-treated with blood transfusions [13]. Being a product of heterogenous sera, ATG recipients who demonstrate hypersensitivity reactions including anaphylaxis can be skin tested prior to ATG administration to aid in determining the hypersensitivity reactions to ATG [14]. Patients who demonstrate hypersensitivity to ATG should not receive this drug unless it is deemed essential and unless the benefits are judged to overweigh the risks. Under such circumstances, these patients may become candidates for ATG desensitization [14].

The adverse effects associated with the use of polyclonal antilymphocyte agents are variable in severity and they include: fever, chills, myalgias, artheralgias, various cutaneous eruptions, serum sickness, gastrointestinal complaints including vomiting and diarrhea, lymphadenopathy, neutropenia, thrombocytopenia, syncope, generalized seizures, disseminated intravascular coagulation, renal dysfunction and hepatotoxicity [6,7,13,15-18]. The adverse effects occur in response to the administration of foreign protein substances but can be prevented by pre-treatment with corticosteroids, diphenhydramine and acetaminophen [7]. Although the adverse effects and the allergic complications associated with the use of ATG may cause substantial morbidity, most of them are usually reversible within few days [6]. To reduce the frequency and the severity of these side effects and complications, it is recommended to: use long intravenous infusion durations over 12 to 18 hours, change the ATG type in subsequent courses of therapy and increase the time interval between repeated cycles of treatment [6,17,19]. Unfortunately antilymphocyte globulin (ALG) and/or ATG may cause prolonged immunodeficiency thus making infectious complications more frequent suggesting the importance of aggressive monitoring of viral and fungal infections [5]. Particular attention should be devoted to the potential of infections with herpes viruses, especially cytomegalovirus, following the use of intensive conditioning regimens particularly those that include ATG [20]. Therefore, ALG/ATG should be used with caution and the negative consequences must be understood and possibly prevented [5]. ATG induction therapy in immunologically high risk individuals induces a profound long-term decrease in the cell counts and the Th1 but not the Th2 responses to CD4+T cells which may explain the long-term effects on infection and the development of post-transplant lymphoproliferative disease because of the inadequate T-cell control [21]. Serum anti-rabbit and/or anti-horse antibodies have been demonstrated in a significant proportion of renal transplant recipients even before transplantation possibly due to an environmental exposure [22].

The adverse effects and the long-term toxicity of anti-lymphocyte agents may vary with the choice of the agent used. Examples include: (1) In certain populations: rabbit ATG has been found to cause more frequent adverse effects than horse ATG, while in other populations, only mild allergic reactions were encountered with the use of rabbit ATG. (2) The anti-human thymocyte globulin form of rabbit ATG has been found to cause significantly greater incidence of cytomegalovirus infections, malignancy and death compared to the anti-human T-lymphocyte immunoserum type of rabbit ATG in renal transplant recipients [4,17,23]. The use of the horse and the rabbit forms of ATG is associated with variable degrees of hepatotoxicity including the elevation of serum ALT level which is usually transient but may persist for about 6 months [2,3,6]. Chronic liver dysfunction is a common complication in long-term BMT survivors [24]. The etiology is often multifactorial with iron overload, chronic hepatitis C infections and chronic GVHD being the main causes [24].

In the patient presented, no immediate allergic reactions were encountered. Also no systemic side effects were observed apart from the isolated and the asymptomatic hepatotoxicity. Both ALT and AST levels became severely elevated after the dose of horse ATG given. Despite the introduction of rabbit ATG, the serum levels of ALT and AST continued to decrease gradually with time. Even serum bilirubin level, which increased moderately in response to the dose of horse ATG given, continued to decrease progressively after the stopping this drug and replacing it with rabbit ATG therapy. Follow up of the patient did not reveal any infectious complications eg cytomegalovirus infection or post-transplant lymphoproliferative disease.

## Conclusion

The antilymphocyte agents play a vital immunosuppressive role in hematopoietic stem cell and solid organ transplantation. The hepatotoxicity associated with the use of ATG varies in severity and in duration. The adverse effects of antithymocyte agents vary with the type of the agent used and they may also vary from one population to another. Therefore, encountering severe side effects and toxicity with one type of ATG should encourage physicians and pharmacists to shift to another kind of ATG particularly in situations where continuation of the drug is essential.

## Competing interests

The author(s) declare that they have no competing interests.

## Authors' contributions

All the authors [hematologists and pharmacists] participated in the management of the patient presented as inpatient during the HSCT hospitalization and as outpatient during her follow up at the HSCT clinic. All the authors read and approved the final form of the manuscript.

## References

[B1] Michallet M-C Saltel F Preville X Flacher M Revillard J-P Genestier L Cathepsin-B-dependent apoptosis triggered by antithymocyte globulins: a novel mechanism of T-cell depletion Blood 2003 102 10 3719 3726 10.1182/blood-2003-04-1075 12893746

[B2] Hanada M Kishimoto Y Nakai K Shimizu T Matsumoto N Miyazaki Y Yamamoto Y Amakawa R Fujimoto M Fukuhara S Antithymocyte globulin treatment in 11 patients with aplastic anaemia Rinsho Ketsueki 2000 41 7 563 567 11020979

[B3] Killick SB Marsh JCW Booth JC Gardon-Smith EC Liver function abnormality following treatment with antithymocyte globulin for aplastic anaemia Bone Marrow Transplant 1997 19 3 249 251 10.1038/sj.bmt.1700653 9028554

[B4] Zimmermann SY Klingebiel T Koehl U Soerensen J Schwabe D Tecelac as antithymocyte globulin in conditioning for childhood allogeneic stem cell transplantation Bone Marrow Transplant 2002 29 12 957 962 10.1038/sj.bmt.1703561 12098062 PMC7091629

[B5] Bacigalupo A Antilymphocyte/thymocyte globulin for graft versus host disease prophylaxis: efficacy and side effects Bone Marrow Transplant 2005 35 3 225 231 10.1038/sj.bmt.1704758 15558041

[B6] Pihusch R Holler E Mühlbayer D Göhring P Stötzer O Pihusch M Hiller E Kolb H-J The impact of antithymocyte globulin on short-term toxicity after allogeneic stem cell transplantation Bone Marrow Transplant 2002 30 6 347 354 10.1038/sj.bmt.1703640 12235518

[B7] Rossi SJ Schroeder TJ Hariharan S First MR Prevention and management of the adverse effects associated with immunosuppressive therapy Drug Saf 1993 9 2 104 131 8397889 10.2165/00002018-199309020-00004

[B8] Gluckman E Auerbach AD Horowitz MM Sobocinski KA Ash RC Bortin MM Butturini A Camitta BM Champlin RE Friedrich W Good RA Gordon-Smith EC Harris RE Klein JP Ortega JJ Pasquini R Ramsay NK Speck B Vowels MR Zhang MJ Gale PP Bone marrow transplantation for Fanconi anemia Blood 1995 86 7 2856 2862 7670120

[B9] Kohli-Kumar M Morris C DeLaat C Sambrano J Masterson M Mueller R Shahidi NT YaniK G Desantes K Friedman DJ Bone marrow transplantation in Fanconi anemia using matched sibling donors Blood 1994 84 6 2050 2054 8081006

[B10] Maschan AA Trakhtman PE Balashov DN Shipicina IP Skorobogatova EV Skvortsova YV Dyshlevaja ZM Samochatova EV Rumiantsev AG Fludarabine, low-dose busulphan and antithmocyte globulin as conditioning for Fanconi anemia patients receiving bone marrow transplantation from HLA-compatible related donors Bone Marrow Transplant 2004 34 4 305 307 10.1038/sj.bmt.1704580 15195080

[B11] Zanis-Neto J Ribeiro RC Medeiros C Andrade RJ Ogasawara V Hush M Magdalena N Friedrich ML Bitencourt MA Bonfim C Pasquini R Bone marrow transplantation for patients with Fanconi anemia: a study of 24 cases from a single institution Bone Marrow Transplant 1995 15 2 293 298 7773221

[B12] Ayas M Al-Jefri A Al-Mahr M Rifai S Al-Seraihi A Tbakhi A Mustafa M Khairy A Mousa E Igbal A Shalaby L El-Solh H Stem cell transplantation for patients with Fanconi anemia with low-dose cyclophosphamide and anti-thymocyte globulins without the use of radiation therapy Bone Marrow Transplant 2005 35 5 463 466 10.1038/sj.bmt.1704787 15654354

[B13] Pawelski S Rokicka-Milewska R Oblakowski P Skwarska E Takiel M Karpowicz M ZdunczyK A Brodzki LM Leszko B ALG/ATG treatment..a useful alternative for BMT in selected aplastic anaemia patients Folia Haematol Int Mag Klin Morphol Blutforsch 1984 116 3-4 377 381 2480277

[B14] Millar MM Grammer LC Case reports of evaluation and desensitization for anti-thymocyte globulin hypersensitivity Ann Allergy Asthma Immunol 2000 85 4 311 316 11061475 10.1016/S1081-1206(10)62535-5

[B15] Steensma DP Dispenzieri A Moore SB Schroeder G Tefferi A Antithymocyte globulin has limited efficacy and substantial toxicity in unselected anemic patients with myelodysplastic syndrome Blood 2003 101 6 2156 2158 10.1182/blood-2002-09-2867 12411290

[B16] Bielory L Yancey KB Young NS Frank MM Lawley TJ Cutaneous manifestations of serum sickness in patients reciving antithymocyte globulin J Am Acad Dermatol 1985 13 3 411 417 3877081 10.1016/s0190-9622(85)70182-x

[B17] Kobayashi R Kaneda M Watanabe N Iguchi A Cho Y Yoshida M Arioka H Naito H Shikano T Ishikawa Y Adverse effects of anti-thymocyte globulin/anti-lymphocyte globulin therapy Rinsho Ketsueki 1999 40 7 531 535 10483134

[B18] Weber M Kröger N Langer F Hansen A Zabelina T Eifrig B Hossfeld OK Zander AR Non-overt disseminated intravascular coagulatin in patients during treatment with antitymocyte globulin for unrelated allogeneic hematopoietic stem cell transplantation Bone Marrow Transplant 2003 31 9 817 822 10.1038/sj.bmt.1703921 12732891

[B19] Ganapiev AA Abdulkadyrov Kal Afanas'ev BV Incidence and characteristics of complication in aplastic anemia patients treated with antilymphocyte globulin Ter Arkh 2003 75 8 68 71 14520856

[B20] Mizue N Watanabe J Katoh S Oda T Suzuki N Kudoh T Unrelated bone marrow transplantation in two severe aplastic anemia patients preconditioned with a regimen of cyclophosphamide, antithymocyte globulin and total body irradiation Rinsho Ketsueki 1999 40 3 213 217 10222628

[B21] Weimer R Staak A Süsal C Steller S Yildiz S Pelzl S Renner F Dietrich H Daniel V Rainer L Kamali-Ernst S Ernst W Padberg W Opelz G ATG induction therapy: long-term effects on Th1 but not Th2 responses Transpl Int 2005 18 2 226 236 10.1111/j.1432-2277.2004.00047.x 15691277

[B22] Prin Mathieu C Renoult E Kennel De March A Béné MC Kessler M Faure GC Serum anti-rabbit and anti-horse IgG, IgA and IgM in Kidney transplant recipients Nephrol Dial Transplant 1997 12 10 2133 2134 10.1093/ndt/12.10.2133 9351078

[B23] Docloux D Kazory A Challier B Coutet J Bresson-Vautrin C Motte G Thalamy B Ribibou JM Chalopin JM Long-term toxicity of antithymocyte globulin induction may vary with choice of agent: a single-centre retrospective study Transplantation 2004 77 7 1024 1033 10.1097/01.TP.0000119163.30745.C1 15087766

[B24] Tomas JF Pinilla I Garcin-Buey ML Garcia A Figuera A Gomez-Garcia de Soria V Moreno R Fernandez-Ranada JM Long-term liver dysfunction after allogeneic bone marrow transplantation: clinical features and course in 61 patients Bone Marrow Transplant 2000 26 6 649 655 10.1038/sj.bmt.1702532 11035372

